# High-Precision Small-Signal Model for Double-Channel–High-Electron-Mobility Transistors Based on the Double-Channel Coupling Effect

**DOI:** 10.3390/mi16020200

**Published:** 2025-02-10

**Authors:** Ziyue Zhao, Qian Yu, Yang Lu, Chupeng Yi, Xin Liu, Ting Feng, Wei Zhao, Yilin Chen, Ling Yang, Xiaohua Ma, Yue Hao

**Affiliations:** 1 The State Key Discipline Laboratory of Wide Band Gap Semiconductor Technology, Faculty of Integrated Circuit, Xidian University, Xi’an 710071, China; zyzhao9303@163.com (Z.Z.);; 2 The School of Advanced Materials and Nanotechnology, Xidian University, Xi’an 710071, China; yl_chen96@163.com

**Keywords:** DC-HEMT, coupling, double-channel coupling sub-model, model

## Abstract

This paper presents a new small-signal model for double-channel (DC)–high-electron-mobility transistors, developed through an analysis of the unique coupling effects between channels in devices. Unlike conventional single-channel HEMTs, where electrons only transport laterally in the channel, DC-HEMTs exhibit additional vertical transport between the two channels along the material direction. This double-channel coupling effect significantly limits the applicability of traditional small-signal models to DC-HEMTs. Firstly, the coupling effect between the two channels is characterized by introducing the double-channel coupling sub-model, which consists of *R*_GaN_, *R*_AlN_, and *C*_AlN_. At the same time, by introducing parameters gm__upper_ and gm__lower_, the new model can accurately characterize the properties of double channels. Secondly, initial values for *R*_GaN_, *R*_AlN_, and *C*_AlN_ are calculated based on the device’s physical structure and material properties. Similarly, initial values for *gm*__upper_ and *gm*__lower_ are derived from the device’s DC measurement and TCAD simulation results. Furthermore, a comprehensive parameter extraction method enables the optimized extraction of intrinsic parameters, completing the model’s construction. Finally, validation of the model’s fitting reveals a significantly reduced error compared to traditional small-signal models. This enhanced accuracy not only verifies the precise representation of the device’s physical characteristics but also demonstrates the model’s effectiveness.

## 1. Introduction

GaN-based high-electron-mobility transistors (HEMTs) have garnered significant attention in recent years due to their superior material properties and device characteristics, which include a wide band-gap, a high breakdown electric field, and high electron saturation velocity [1,2,3,4,5]. Furthermore, it has found applications in radar detection and mobile communications. With the development of next-generation communication technologies, GaN HEMTs present broader opportunities for application [6,7]. Meanwhile, these applications also impose stricter demands on the output power density, efficiency, and linearity of GaN HEMTs.

Despite numerous studies reporting the excellent characteristics of GaN HEMTs, the exponential increase in source resistance (*R*_s_) and the poor saturation velocity (*V*_SAT_) under high-frequency, large-signal conditions lead to a significant reduction in output current swing, resulting in a marked decline in device linearity [8,9].

At present, Fin-gate structures and graded channels are commonly employed to enhance the linearity of GaN HEMTs [10,11,12,13]. The Fin-HEMT improves device linearity by increasing the effective control area of the gate. However, this design also reduces the effective gate width, which decreases the saturation output current. Graded channels enhance gm flatness and linearity by compensating between the recess and non-recess regions. Nevertheless, this approach can also lead to reductions in both saturation current and efficiency. Both methods struggle to achieve simultaneous improvements in device linearity and output power. Recently, extensive research has focused on the AlGaN/GaN double-channel (DC)–HEMT structure [14,15]. By utilizing double channels and high-current drive, this approach addresses the trade-off between output power and linearity inherent in traditional device structures [16,17]. The DC-HEMT redistributes carriers between the two channels, thereby improving the *R*_s_–*I*_ds_ relationship and increasing the *V*_SAT_. Additionally, DC-HEMT transforms carrier distribution from 2DEG to 3DEG, reducing optical phonon scattering. Consequently, double channels facilitate a gradual decline in transconductance (gm) and significantly improve the linearity of the device. Furthermore, the double-channel structure supports higher current drive, which effectively increases the device’s saturation output current, leading to greater output power.

Currently, research on DC-HEMTs primarily focuses on the analysis of device materials, structures, and both direct current and radio frequency characteristics. Studies on equivalent circuit models for DC-HEMTs are relatively scarce. However, models are central to bridging the gap between device and engineering applications. A well-designed model can effectively facilitate the implementation of new structural devices in engineering [18]. Nevertheless, there has been little research on the use of small-signal modeling (SSM) for DC-HEMTs, which is essential for analyzing parameter behavior, optimizing processes, and improving update efficiency [19]. In addition, the SSM is the foundation of the large-signal model. The SSM can determine the topology of the device and the nonlinear trend of the intrinsic parameters of the device, which is also crucial for the establishment of the large-signal model.

This paper investigates the coupling mechanism between channels in an independently developed DC-HEMT based on device structure and operational principles. It introduces the double-channel coupling sub-model (DCC-SM) and develops a high-precision small-signal model. Initial parameter values are extracted using the device’s structural characteristics and TCAD simulations, improving the accuracy and efficiency of parameter extraction. Furthermore, the paper presents a complete parameter extraction method to facilitate the model’s application. Section 2 details the DC-HEMT fabrication process, while Section 3 discusses the measurement of its DC and RF characteristics, followed by an analysis of the double-channel coupling effect. Section 4 explores the coupling mechanism, establishes the DCC-SM, and provides a complete parameter extraction method. Section 5 presents model fitting results, confirming the accuracy and effectiveness of the proposed model.

## 2. Device Structures and Process

**Figure 1:**
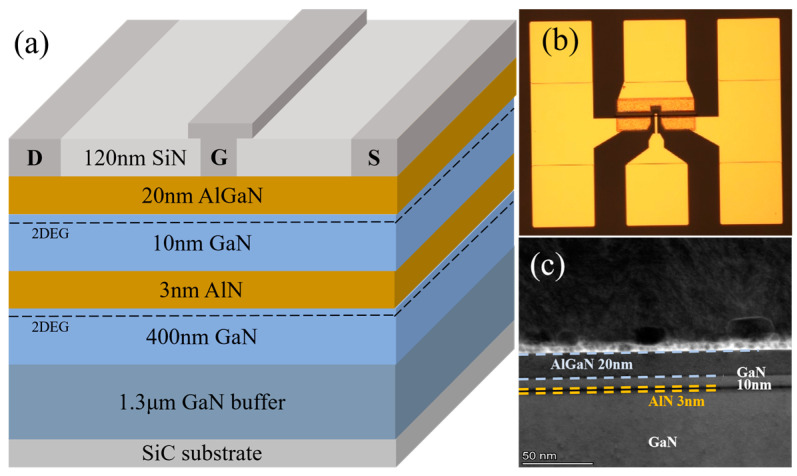
(**a**) The structure diagram of the DC-HEMT. (**b**) The optical photoscope photograph of the device. (**c**) The TEM of the device.

The DC-HEMT used for research was independently developed by Xidian University. Figure 1a presents a structure diagram of the DC-HEMT. The device was fabricated using metal–organic chemical vapor deposition (MOCVD) on a 3-inch 4H-SiC substrate. The epitaxial layers, arranged from bottom to top, include a 1.3 μm Fe-doped GaN buffer layer, a 400 nm unintentionally doped GaN channel layer, a 3 nm AlN barrier layer, a 10 nm GaN sub-channel layer, and a 20 nm AlGaN barrier layer. To enhance this coupling effect, a 3 nm ultra-thin barrier layer and a 10 nm upper GaN sub-channel layer are employed.

The fabrication of the device begins with the Ohmic contact. A Ti/Al/Ni/Au metal stack is deposited and annealed at 850 °C for 50 s in a nitrogen (N_2_) atmosphere. Planar isolation is achieved through nitrogen ion implantation, and the device is passivated with a 120 nm SiN_X_ layer deposited through PECVD. CF_4_-based plasma etching removes the SiN_X_ layer to expose the gate foot, and the T-gate cap is defined through photolithography. Subsequently, Ni/Au Schottky contacts for the gate are deposited through electron-beam evaporation. Finally, the interconnects of the device are formed using a Ti/Au metal stack. Upon completion of the above steps, the device’s fabrication is finalized. The source-drain spacing (*L*_sd_) and gate length (*L*_g_) are 5 μm and 0.5 μm, respectively. The device features a two-finger structure, with each finger having a gate width (*W*_g_) of 50 μm. An optical photoscope photograph of the device is shown in Figure 1b. To verify the capability of the process, the transmission electron microscope (TEM) of the device is shown in Figure 1c. As can be seen from the figure, the ultra-thin AlN barrier has a high quality.

## 3. Measurement and Analysis

**Figure 2:**
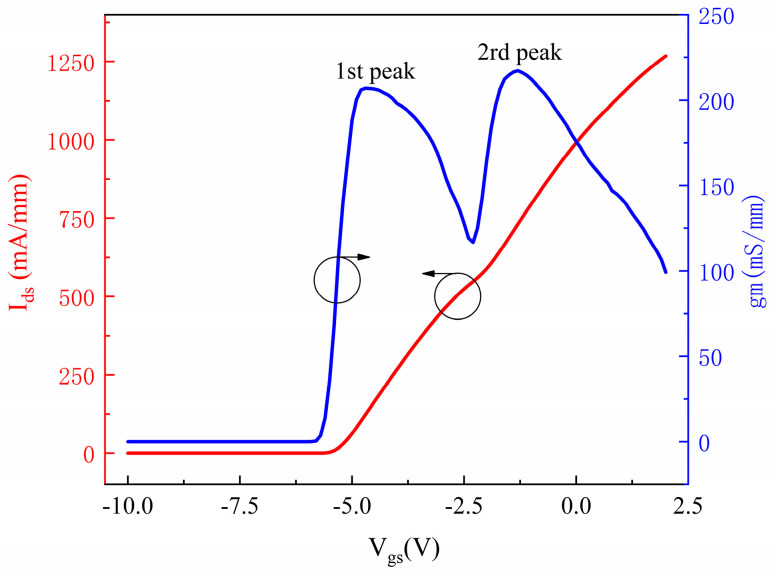
The transfer characteristics of the DC-HEMTs. The *V*_gs_ was varied from −10 V to 2 V, and the *V*_ds_ was set to 10 V. The current curve is represented by the red curve, and the transconductance curve is represented by the blue curve.

The DC characteristics of the DC-HEMT were characterized using the Keysight B1500A. The gate-source voltage (*V*_gs_) was varied from −10 V to 2 V in 0.1 V steps, while the drain-source voltage (*V*_ds_) was set to 10 V. The transfer characteristics of the device are presented in Figure 2. From the drain-source current (*I*_ds_) curve, it is observed that when the *V*_gs_ is 2 V, the maximum drain current density reaches 1268 mA/mm. The high current density demonstrates the significant advantage of DC-HEMTs in improving current drive capability. In Figure 2, it is evident that the threshold voltage (*V*_th_) of the device is −5.9 V, and the gm curve exhibits a distinct double-peak characteristic. The first and second peaks of gm are 207 mS/mm and 217.2 mS/mm, and the corresponding gate voltages are −4.7 V and −1.3 V, respectively. The second peak is slightly higher than the first one. When *V*_gs_ is −10 V, the device is in a completely off state. As *V*_gs_ increases, the lower channel gradually turns on. When *V*_gs_ reaches −4.7 V, the gm of the lower channel attains its maximum value, marking the first peak of gm. As *V*_gs_ continues to increase, the carrier concentration in the lower channel approaches saturation. When *V*_gs_ exceeds −2 V, the upper channel begins to turn on, and the gm corresponding to the upper channel gradually increases while the gm corresponding to the lower channel decreases to zero. When *V*_gs_ reaches −1.3 V, gm reaches the second peak. Subsequently, as *V*_gs_ increases, the Ids of the device gradually saturates, and gm gradually decreases.

Theoretically, the second peak of gm is significantly higher than the first peak due to the proximity of the upper channel to the gate and the thinner barrier layer of the device. However, as shown in Figure 2, the values of the two peaks are relatively close. This phenomenon is attributed to the double-channel coupling effect between the upper and lower channels of the DC-HEMTs.

**Figure 3:**
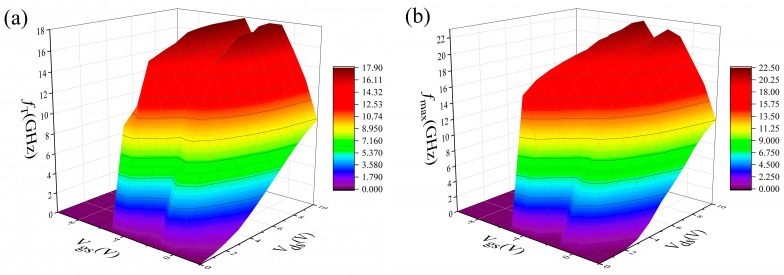
(**a**) The f_T_ of the DC-HEMT. (**b**) The *f*_max_ of the DC-HEMT. The *V*_gs_ was varied from −10 V to 2 V, and the *V*_ds_ was varied from 0 V to 10 V.

The S-parameters of the device were characterized using the Keysight E8363B under various biases. First, the S-parameters can characterize the device’s frequency characteristics, allowing for the determination of *f*_T_ and *f*_max_ of the device. Second, the S-parameters enable the establishment of a small-signal model of the device. The *V*_gs_ of the device ranges from −10 V to 2 V, while the V_ds_ varies from 0 V to 10 V, with a step size of 1 V. The frequency range extends from 0.1 GHz to 30 GHz. The *f*_T_ and *f*_max_ of the device under various biases are shown in Figure 3. Figure 3a shows the *f*_T_ of the DC-HEMT, exhibiting a distinct double-peak characteristic as gate biases. According to the formula [14, 20] for *f*_T_, which is shown in (1), it is positively correlated with gm and negatively correlated with gate capacitance (*C*_g_). This relationship explains why the shape of *f*_T_ is consistent with the gm. However, unlike the gm curve, when the drain voltage of the device remains constant, the first peak of the *f*_T_ is higher than the second peak. This phenomenon occurs because although the second peak of the gm is greater than the first peak, the difference between the two peaks is small. When the upper channel is off, the *C*_g_ of the device is significantly smaller than when the upper channel is on. Consequently, the first peak of the *f*_T_ is higher than the second peak.

(1) $$f_{T}\approx \frac{gm}{2\pi C_{g}},$$

(2) $$f_{\max}\approx \frac{f_{T}}{2\sqrt{\frac{R_{i}+R_{s}+R_{g}}{R_{ds}}+(2\pi f_{T})R_{g}C_{gd}}},$$

Similarly, it can be inferred from the formula in [20] that *f*_max_ is positively correlated with *f*_T_ and negatively correlated with gate-drain capacitance (*C*_gd_). Therefore, when the upper channel is turned off, the device exhibits a higher *f*_T_ and a lower *C*_gd_, resulting in a larger *f*_max_ in this state. As the *V*_gs_ increases, the decrease in *f*_T_ and the increase in *C*_gd_, combined with the reduction in *R*_ds_ resulting from the upper channel turning on, all contribute to the degradation of *f*_max_. Therefore, the first peak of *f*_max_ will also be higher than the second peak of the *f*_max_.

## 4. The New Small-Signal Model

### 4.1. Double-Channel Coupling Effect

**Figure 4:**
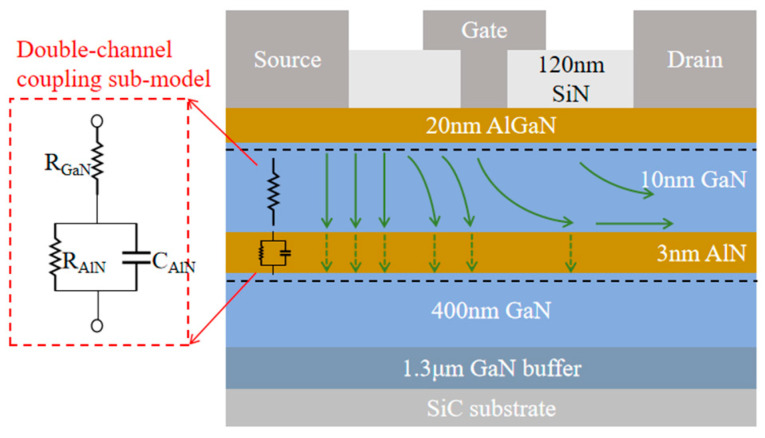
Electron transport mechanism between two GaN channel layers, and the establishment of the double-channel coupling sub-model.

Compared to traditional small-signal models, the DC-HEMT model not only requires separate characterization of the upper and lower channels (through gm__upper_ and gm__lower_); more importantly, it must account for the impact of double-channel coupling effects. The coupling modulation between the channels fundamentally alters the electron transport mode in the upper channel, and the modulation mechanism is illustrated in Figure 4 [14], which is verified by the TCAD simulation. 

For DC-HEMTs, electron transport between the two channels is primarily vertical, with lateral transport also occurring in the GaN sub-channel layer. Therefore, based on the electron transport characteristics, a DCC-SM has been established, as illustrated in Figure 4. R_AlN_ is introduced to characterize the vertical transport of electrons in the AlN insertion layer, while resistance R_GaN_ is introduced to represent the vertical transport of electrons in the GaN sub-channel layer. Simultaneously, considering that electrons undergo lateral transport in the sub-channel layer, forming a capacitance characteristic with the 2DEG of the lower channel, capacitance C_AlN_ is introduced to characterize this capacitive effect. Additionally, C_AlN_ forms an RC parallel network with R_AlN_. Ultimately, by introducing the parameters R_GaN_, R_AlN_, and C_AlN_, as shown in Figure 4, the double-channel coupling effect of the DC-HEMT is characterized, while the RC parallel network represents the delay characteristics induced by bulk traps.

The initial value of the parameter can be calculated based on the physical size of the device. These results serve as reliable initial values for the model. Considering that a quantum well is formed in the GaN sub-channel layer and that the thickness of the GaN sub-channel layer is 10 nm, we simplify the 10 nm GaN layer into two regions: a 2 nm quantum well region and an 8 nm GaN bulk material region. The resistivity of the 8 nm GaN bulk material region is calculated using the following formula:

(3) $$\rho_{GaN}=\frac{1}{q\cdot \mu_{n}\cdot n},$$

In (3), *q* represents the charge of an electron, *μ*_n_ denotes the electron mobility, and n indicates the electron concentration. Because the coupling current flows along the material direction, *μ*_n_ refers to the electron mobility in bulk GaN, which is 1200 cm^2^/V∙s. The variation of the carrier concentration with depth is obtained through capacitance-voltage (C-V) testing [14], and the average carrier concentration n for the upper channel and the lower barrier layer is considered to be 1 × 10^18^ cm^−3^.

The *L*_sd_ of the device is 5 μm, and the *W*_g_ of the device is 2 × 50 μm. Consequently, the resistance value *R*_GaN_ can be calculated using Formula (4).

(4) $$R_{GaN}=\rho \frac{L}{S}=\rho_{GaN}\frac{h_{GaN}}{L_{sd}\cdot W_{g}}=8\times 10^{-4}\Omega ,$$

Because the resistance value does not consider the influence of the quantum well in the GaN sub-channel layer, the result of Formula (4) will be smaller than the actual value. However, it is only used as an initial value, and subsequent parameter optimization effectively eliminates the error caused by this simplification. Moreover, the actual value of the parameter will not be smaller than its initial value.

Similarly, the initial value of the R_AlN_ is approximately 2.5 × 10^−2^ Ω.

The dielectric constant of the AlN is about 8.5. Applying the parallel plate capacitor formula, the initial value of the parameter C_AlN_ is approximately 1.28 × 10^−11^ F.

During the actual operation of the device, the effective area for electron tunneling is smaller than the theoretical value, leading to an increase in the coupling resistances R_AlN_ and R_GaN_, and the actual area is related to the device’s biases. When the 2DEG in the lower channel is determined based on the specific bias, the coupling capacitance C_AlN_ becomes smaller than the initial value. Errors in the coupling parameters can be corrected and eliminated through optimization algorithms. Following these steps allows for the completion of the double-channel coupling sub-model and the extraction of the initial values.

**Figure 5:**
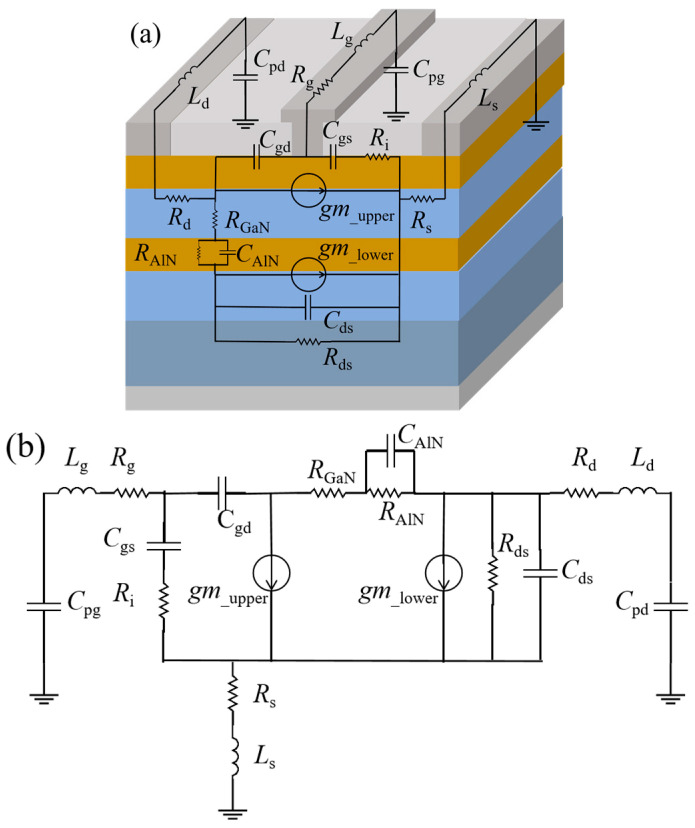
(**a**) The stereogram of the DC-HEMT and the structure of the SSM. (**b**) The equivalent circuit topology of the DC-HEMT.

After establishing the DCC-SM and combining it with the *gm*__upper_ and *gm*__lower_, a complete SSM of the device is constructed based on its structure, as illustrated in Figure 5a. This model incorporates eight parasitic parameters, representing the parasitic capacitances, inductances, and resistances of the gate, the drain, and the source, which are represented by *C*_pg_, *C*_pd_, *L*_g_, *L*_d_, *L*_s_, *R*_g_, *R*_d_, and *R*_s_, consistent with the traditional small-signal model of GaN HEMTs.

Compared to the traditional SSM, the new DC-HEMT model introduces *gm*__upper_ and *gm*__lower_ to represent the upper and lower channels, respectively. As shown in Figure 5a, the DCC-SM is incorporated between *gm*__upper_ and *gm*__lower_ to characterize the double-channel coupling effects between the channels. For more clarity, the topology of the small-signal model is illustrated in Figure 5b. The next step involves extracting the value of intrinsic parameters.

### 4.2. Device Simulation Results

**Figure 6:**
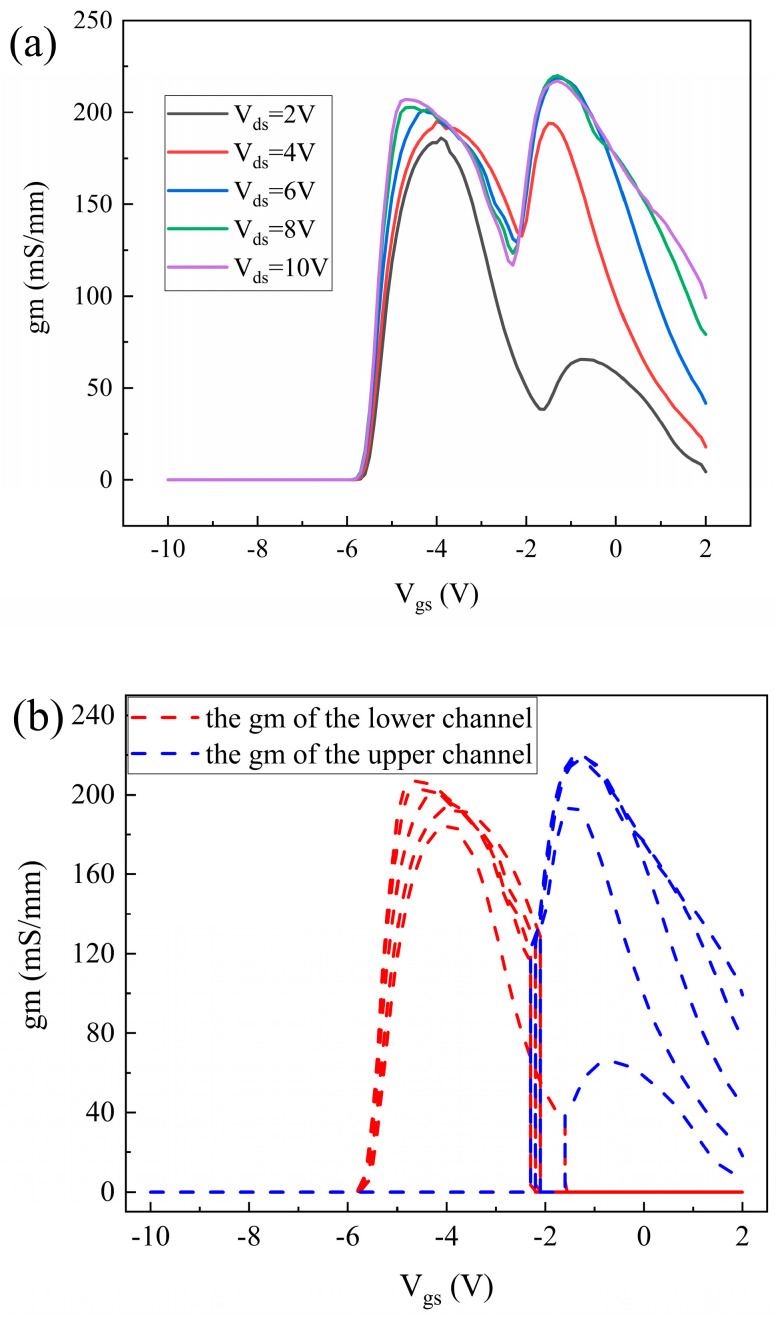
(**a**) Transconductance curve in DC-HEMT with different drain voltages. (**b**) Transconductance curve of the lower channel and the upper channel.

To further simplify parameter extraction, gm curves at various biases are utilized for the preliminary characterization of *gm*__upper_ and *gm*__lower_, as shown in Figure 6a. The figure demonstrates that the gm curves exhibit a double-peak characteristic at different *V*_ds_, with a distinct transition point between the two peaks. It can be inferred that when the *V*_gs_ is below the transition point, only the lower channel is turned on, while the upper channel remains in an off state. At this point, the drain current is entirely supplied by the lower channel, meaning a gm value of zero for the upper channel. When the *V*_gs_ exceeds the transition point, the lower channel saturates, and the upper channel activates. The current of the upper channel is equal to the total drain current of the device minus the drain current at the transition point. Consequently, the gm value for the lower channel is zero. The gm curves for the upper and lower channels are presented in Figure 6b. The gm curves of the upper channel are represented by blue lines, and the gm curves of the lower channel are represented by red lines. This method can only qualitatively characterize the trends in the gm values of the upper and lower channels, and it cannot accurately quantify these values, which may hinder the improvement of fitting accuracy for the SSM.

**Figure 7:**
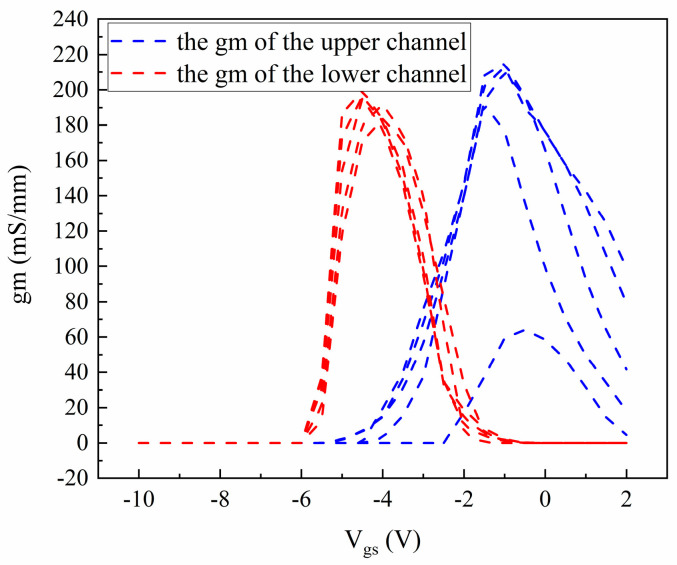
Transconductance curve of the lower channel and the upper channel considering the simulation result of the Silvaco.

In the actual operating state of the device, the gm does not increase or decrease sharply with the V_gs_ but exhibits an overlap at the transition point. To address this issue, the characteristics of gm are simulated using Technology Computer-Aided Design (TCAD, by Silvaco). First, the physical model is established based on the structure shown in Figure 1. Because the upper channel is closer to the gate and its operating principle is similar to that of a conventional single-channel HEMT, the simulation structure is relatively simple, allowing for accurate fitting of the upper channel layer. However, due to the presence of dielectric layers (the upper barrier layer and the sub-channel layer) between the lower channel and the gate, the simulation structure becomes complex, leading to significant errors in the simulation. To ensure the accuracy of the extraction of the gm for the lower channel, the gm characteristics of the lower channel are fitted by subtracting the simulated gm of the upper channel from the measured gm. The resulting gm is illustrated in Figure 7. It is important to note that the simulation model does not accurately capture the double-channel coupling characteristics. Therefore, the curves presented in Figure 6b fail to accurately characterize the gm characteristics of both the upper and lower channels. However, these values can serve as initial values in the small-signal model, facilitating accurate parameter extraction while enabling the correction of errors through optimization algorithms.

### 4.3. Extracting Model Parameters

**Figure 8:**
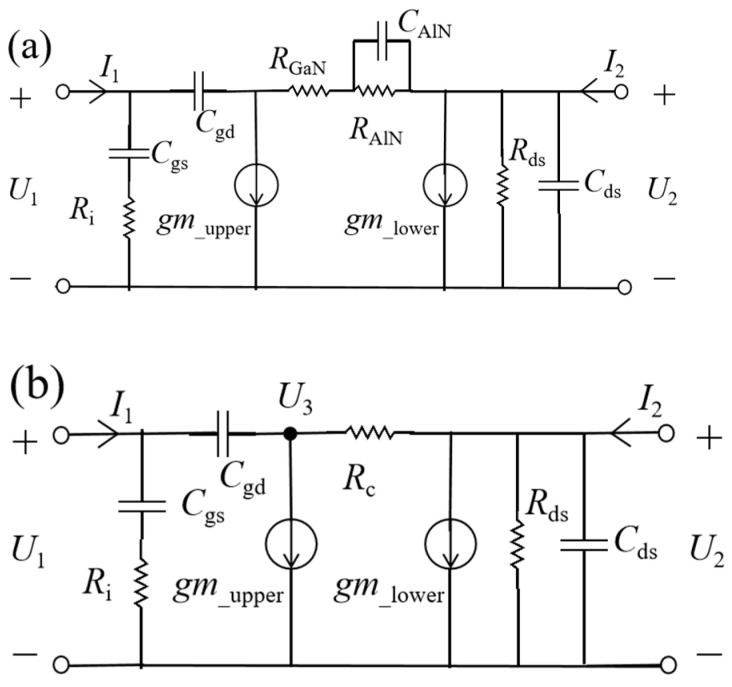
(**a**) The topology diagram of the intrinsic region of the DC-HEMT. (**b**) The topology diagram of the intrinsic region of the DC-HEMT, where the DCC-SM is simplified.

The extraction of parameters primarily relies on measuring S-parameters under various biases. Based on the topology presented in Figure 5b, the parasitic parameters of the model can be extracted and de-embedded using the Cold-FET method [21,22,23], obtaining the intrinsic Y-parameters. The configuration of the intrinsic parameters is illustrated in Figure 8a.

The topological structure in Figure 5b is more complex than traditional models. To obtain intrinsic parameters quickly and accurately, further simplification and analysis of the device’s topology are required. 

First, the double-channel coupling sub-model, consisting of *R*_GaN_, *R*_AlN_, and *C*_AlN_, is analyzed. The impedance characteristics of the sub-model ((R_c_ + jX_c_) Ω) are represented by Formula (5).

(5) $$\begin{array}{ll} R_{c}+jX_{c} & =R_{GaN}+\frac{1}{1/R_{AlN}+j\omega C_{AlN}}\\ & =R_{GaN}+\frac{R_{AlN}}{1+\omega^{2}R_{AlN}^{2}C_{AlN}^{2}}-j\omega \frac{R_{AlN}C_{AlN}}{1+\omega^{2}R_{AlN}^{2}C_{AlN}^{2}} \end{array},$$

From the previous sections, the initial values of *R*_GaN_, *R*_AlN_, and *C*_AlN_ can be determined. Then,

(6) $$\omega^{2}R_{AlN}^{2}C_{AlN}^{2}\ll 1,$$

So,

(7) $$1+\omega^{2}R_{AlN}^{2}C_{AlN}^{2}≅1,$$

(8) $$R_{c}=R_{AlN}+R_{GaN},$$

(9) $$X_{c}=-\omega R_{AlN}C_{AlN},$$

Additionally, because the value of *C*_AlN_ is known and X_c_ ≈ 0, further simplification of the intrinsic topology is performed, as shown in Figure 8b.

To simplify the analysis of the entire network structure, a potential reference point, U_3_, is introduced. Thus, the following can be obtained:

(10) $$I_{1}=\frac{j\omega C_{gs}}{1+j\omega R_{i}C_{gs}}U_{1}+j\omega C_{gd}(U_{1}-U_{3}),$$

(11) $$I_{2}=gm_{\_lower}U_{1}+(j\omega C_{ds}+\frac{1}{R_{ds}})U_{2}+\frac{1}{R_{c}}(U_{2}-U_{3}),$$

Additionally, using Kirchhoff’s current law, the relationship between *U*_1_, *U*_2_, and *U*_3_ can be obtained.

(12) $$j\omega C_{gd}(U_{1}-U_{3})=gm_{\_upper}U_{1}+\frac{1}{R_{c}}(U_{3}-U_{2}),$$

Therefore, the following can be obtained:

(13) $$U_{3}=\frac{j\omega C_{gd}-gm_{\_upper}}{j\omega C_{gd}+1/R_{c}}U_{1}+\frac{1}{R_{c}(j\omega C_{gd}+1/R_{c})}U_{2},$$

The Y-parameters of the intrinsic network can be expressed as follows:

(14) $$\begin{array}{ll} Y_{\mathrm{int}11} & =\frac{\omega^{2}R_{i}C_{gs}^{2}}{1+\omega^{2}R_{i}^{2}C_{gs}^{2}}-\frac{\omega^{2}C_{gd}^{2}+gm_{\_upper}g_{c}}{g_{c}^{2}+\omega^{2}C_{gd}^{2}}\\ & +j(\frac{\omega C_{gs}}{1+\omega^{2}R_{i}^{2}C_{gs}^{2}}+\omega C_{gd}\frac{g_{c}^{2}+\omega^{2}C_{gd}^{2}-gm_{\_upper}-g_{c}}{g_{c}^{2}+\omega^{2}C_{gd}^{2}}) \end{array},$$

(15) $$Y_{\mathrm{int}12}=\frac{g_{c}^{2}}{g_{c}^{2}+\omega^{2}C_{gd}^{2}}-j\omega \frac{C_{gd}g_{c}}{g_{c}^{2}+\omega^{2}C_{gd}^{2}},$$

(16) $$\begin{array}{ll} Y_{\mathrm{int}21} & =gm_{\_lower}-\frac{\omega^{2}C_{gd}^{2}+gm_{\_upper}g_{c}}{g_{c}^{2}+\omega^{2}C_{gd}^{2}}\\ & -j\omega C_{gd}\frac{gm_{\_upper}+g_{c}}{g_{c}^{2}+\omega^{2}C_{gd}^{2}} \end{array},$$

(17) $$\begin{array}{cc} Y_{\mathrm{int}22} & =g_{c}+g_{ds}-\frac{g_{c}^{3}}{g_{c}^{2}+\omega^{2}C_{gd}^{2}}\\ & +j\omega (C_{ds}+\frac{C_{gd}}{g_{c}^{2}+\omega^{2}C_{gd}^{2}}) \end{array},$$

The value of *C*_gd_^2^ can be obtained from the slope between 1/real(*Y*_int12_) and ω^2^.

Because ω^2^*R*_i_^2^*C*_gs_^2^ is significantly less than 1 (for frequencies below 5 GHz), it can be approximated as 1 + ω^2^*R*_i_^2^*C*_gs_^2^ ≈ 1, and then

(18) $$imag(Y_{\mathrm{int}11})\approx \omega (C_{gs}+C_{gd})-\frac{gm_{\_upper}+g_{c}}{g_{c}^{2}},$$

The value of *C*_gs_ can be obtained from the slope between imag(*Y*_int11_) and ω.

Using the same method, the following can be obtained:

(19) $$real(Y_{\mathrm{int}11})\approx \omega^{2}(R_{i}C_{gs}^{2}+\frac{C_{gd}^{2}}{g_{c}^{2}})+\frac{gm_{\_upper}}{g_{c}},$$

The value of R_i_ can be obtained from the slope between real(*Y*_int11_) and ω^2^.

(20) $$real(Y_{\mathrm{int}22})\approx g_{ds},$$

(21) $$imag(Y_{\mathrm{int}22})\approx \omega (C_{ds}+\frac{C_{gd}}{g_{c}^{2}})$$

Because *C*_gd_ and *g_c_* are known, *C*_ds_ can be obtained from the slope between imag(*Y*_int22_) and ω.

**Figure 9:**
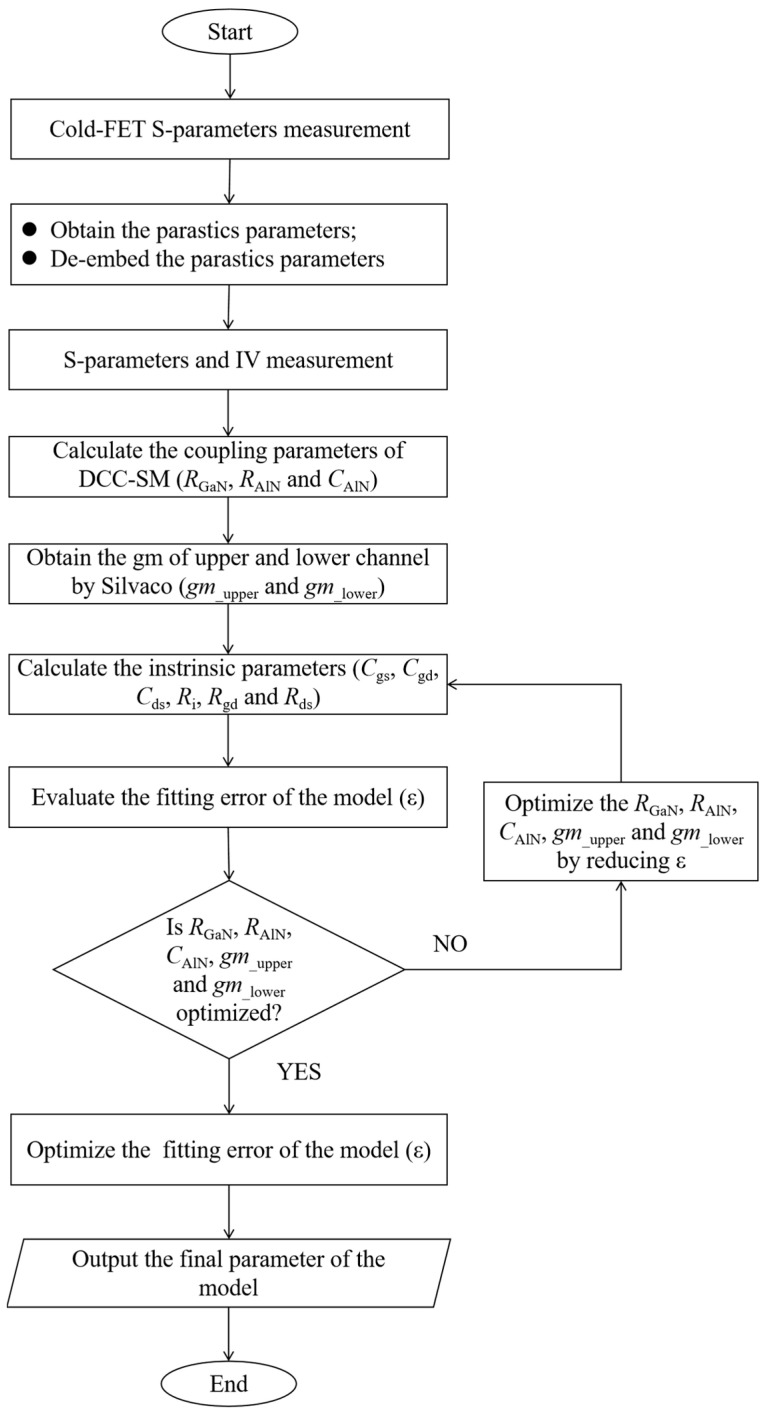
Flowchart of the model’s parameter value generation algorithm.

Utilizing the calculations from the aforementioned method, the initial values of the intrinsic parameters can all be determined. Given that there are inherent errors in the double-channel coupling parameters and the gm values, it is essential to optimize the parameters *R*_GaN_, *C*_AlN_, *R*_AlN_, *gm*__upper_, and *gm*__lower_. The gradient optimization algorithm in ADS is selected for optimization. Based on the optimized results, the intrinsic parameter values will be re-extracted. Ultimately, parameter extraction for the new model is completed. The flowchart illustrating the model parameter extraction process is presented in Figure 9. Environmental temperature, device dimensions, and material properties influence the values of the model parameters but do not alter its topology. The topology of the DCCSM is fundamentally determined by its operating mechanism.

## 5. Results

To validate the effectiveness of the new model, the parameters of the DC-HEMT are extracted and modeled using biases of (−1 V, 8 V) and (−3 V, 6 V) as examples. The parameter values are presented in Table 1. These two bias states correspond to the device being in a single lower channel in on-state and a double-channel in on-state, respectively. To further validate the effectiveness of the new model, this study also models the DC-HEMT using the traditional model in [18] by comparing the fitting results of the traditional model with the new model proposed in this paper. The topology of the model in [21] is the typical model for GaN HEMT. Due to the advanced method of model parameter extraction, the fitting accuracy of the model in [18] is mainly limited by the topology’s structure, so it can be used as the comparison for DCCSM.

**Figure 10:**
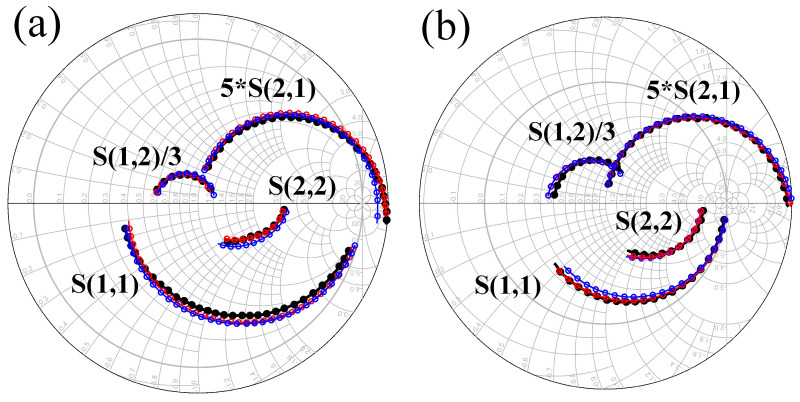
The S-parameter fitting results of the new model and the traditional model. (**a**) The bias state is (−1 V, 8 V). (**b**) The bias state is (−3 V, 6 V). The black curve represents the measured S-parameter values, the red curve denotes the fitting values of the new model with DCC-SM, and the blue curve indicates the fitting values of the traditional model presented in [18].

The fitting results of the S-parameters under both biases are shown in Figure 10. The black curve represents the measured S-parameter values, the red curve denotes the fitting values of the new model with DCC-SM, and the blue curve indicates the fitting values of the traditional model presented in [18]. The fitting errors of the models are calculated using the method in [21], and the results are shown in Table 2. The ε(S_11_), ε(S_12_), ε(S_21_), and ε(S_22_) represent the fitting error of the S_11_, S_12_, S_21_, and S_22_ of the model. It is evident that the new model significantly enhances the fitting accuracy of the S-parameters, particularly for S_12_ and S_21_. This improvement in S-parameter fitting accuracy provides evidence for the scientific validity of the new model with DCC-SM. The improvement in the fitting accuracy of the device’s S-parameters further demonstrates the advancement of the device’s topology. It also ensures the precise extraction of intrinsic capacitances under various bias conditions, thereby enhancing the accuracy of the subsequent large-signal modeling of the DC-HEMT. 

## 6. Conclusions

This paper establishes a double-channel coupling sub-model based on the coupling characteristics between the upper and lower channels and develops a small-signal model for DC-HEMTs. To enable effective parameter extraction, TCAD simulations are conducted to obtain the transconductance values corresponding to the upper and lower channels under different bias conditions. Additionally, considering the material and structural characteristics of the device, the initial values of *R*_GaN_, *R*_AlN_, and *C*_AlN_ in the sub-model are calculated. Finally, parameter optimization is performed to eliminate fitting errors caused by inaccuracies in the initial values, yielding the final parameter values. To verify the accuracy and effectiveness of the proposed model, the fitting performance of the model is evaluated under multiple biases and compared with traditional models. The results demonstrate that the DCC-SM effectively captures the coupling behavior between the device channels, improving the parameter fitting accuracy of DC-HEMTs. The accurate establishment of the small-signal model also lays the foundation for the subsequent development of the large-signal model. Furthermore, this model has significant potential for advancing the application of DC-HEMTs in circuit design and engineering.

## Figures and Tables

**Table 1:** Intrinsic parameters of the device under (−1 V, 8 V) and (−3 V, 6 V).

Parameters	(V_gs_ = −1 V, V_ds_ = 8 V)	(V_gs_ = −3 V, V_ds_ = 6 V)
*C*_gs_ (F)	2.85 × 10^−13^	1.14 × 10^−13^
*C*_gd_ (F)	8.68 × 10^−14^	5.57 × 10^−14^
*C*_ds_ (F)	1.99 × 10^−15^	1.71 × 10^−14^
*R*_i_ (Ω)	14.86	5.61
*R*_gd_ (Ω)	21.24	12.93
*g*ds (S)	7.15 × 10^−3^	2.51 × 10^−3^
*gm*__upper_ (S)	9.58 × 10^−3^	7.49 × 10^−3^
*gm*__lower_ (S)	1.86 × 10^−3^	6.93 × 10^−2^
*R*_GaN_ (Ω)	6.12	1.53
*R*_AlN_ (Ω)	14.88	2.17
*C*_AlN_ (F)	10.01 × 10^−14^	9.03 × 10^−12^

**Table 2:** The fitting errors of the new model and the traditional model.

Error(ε)	(*V*_gs_ = −1 V, *V*_ds_ = 8 V)		(*V*_gs_ = −3 V, *V*_ds_ = 6V)	
	The New Model	The Traditional Model [18]	The New Model	The Traditional Model [18]
ε(S_11_)	1.61%	4.12%	1.92%	3.28%
ε(S_12_)	1.32%	4.31%	3.35%	4.27%
ε(S_21_)	3.10%	9.68%	4.73%	8.42%
ε(S_22_)	2.41%	2.64%	1.71%	2.15%

## Data Availability

The original contributions presented in the study are included in the article, further inquiries can be directed to the corresponding author.
